# Seroprevalence of Pandemic Influenza Viruses, New York, New York, USA, 2004

**DOI:** 10.3201/eid1811.120156

**Published:** 2012-11

**Authors:** Isaac B. Weisfuse, Tshidi Tsibane, Kevin J. Konty, Joseph R. Egger, Elizabeth Needham Waddell, Saad Rahmat, Emily Harris, Donald R. Olson, Christopher F. Basler

**Affiliations:** New York City Department of Health and Mental Hygiene, New York, New York, USA (I.B. Weisfuse, K.J. Konty, J.R. Egger, E.N. Wadell, D.R. Olson);; Mount Sinai School of Medicine, New York (T. Tsibane, S. Rahmat, E. Harris, C.F. Basler);; and International Society for Disease Surveillance, New York, (D.R. Olson)

**Keywords:** influenza, pandemic influenza, seroprevalence, New York, pandemic, influenza A(H1N1)pdm09, susceptibility, immunity, viruses, New York City, United States

**To the Editor:** Exposures to influenza viruses can lead to immune responses that substantially affect susceptibility to infection with related viruses. Characterization of preexisting immunity within a population can inform public health, as highlighted during the influenza A(H1N1)pdm09 virus pandemic, when surveillance data demonstrated that older persons (>65 years old) were less likely than younger persons to have influenza (*1*). Seroprevalence studies of prepandemic samples show that older persons had preexisting antibody responses to A(H1N1)pdm09 virus, presumably because of prior exposure to related strains (*2*). The A(H1N1)pdm09 virus possesses hemagglutinin and neuraminidase genes derived from classical swine influenza virus (*3*).

Epidemiologic and molecular data indicate that prior exposure to early twentieth century H1N1 viruses conferred immunity to A(H1N1)pdm09 virus. Human antibodies that neutralize A(H1N1)pdm09 virus and H1N1 subtype viruses from earlier in the twentieth century have been characterized, and animal studies have demonstrated that antibodies to the earlier H1N1 subtype viruses cross-neutralize A(H1N1)pdm09 virus and protect from virus challenge (*2*,*4*–*6*). Prior exposure to antigenically related viruses can explain the relationship between age and susceptibility to infection.

To determine the seroprevalence of preexisting hemagglutinin inhibition (HAI) antibody titers to influenza strains with pandemic potential, we tested serum samples for antibodies to A(H1N1)pdm09 virus and the 1918, 1957, and 1968 pandemic viruses. The samples had been collected in 2004 from a representative sample of adults in New York City (NYC), USA, as part of the NYC Health and Nutrition Examination Survey (Technical Appendix). For the 1918 and A(H1N1)pdm09 viruses, the highest prevalence of HAI titers >40 was among persons born before 1940 (>65 years old in 2004), although younger adults also had antibodies. Antibody prevalence to the 1957 H2N2 subtype virus was highest among persons born during 1942–1961, and >70% in persons born before 1971 had antibody to the 1968 H3N2 subtype virus (Figure). For all pandemic viruses, there was no significant difference in seroprevalence by sex or by US birth and only minor differences by race/ethnicity (Technical Appendix Table 1).

**Figure:**
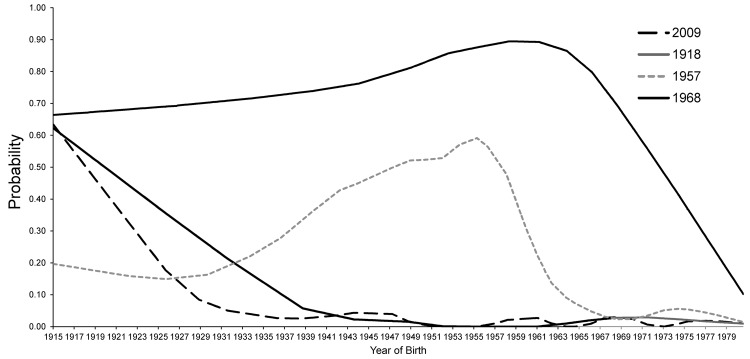
Seroprevalence of cross-reactive antibodies to the 1918, 1957, 1968, and 2009 pandemic influenza viruses among persons >23 years of age, New York, New York, 2004. LOESS (locally weighted scatterplot smoothing) curves represent the estimated prevalence of hemagglutination-inhibition antibody titers of >40 (positive titers) by year of birth.

We examined A(H1N1)pdm09 virus seroprevalence by the age of persons tested and by antibody titer. The mean age for persons with no serologic evidence of prior exposure (titer <20) was 50 years, compared with 72 years for those with titers of 20–40 and 80 years for those with titers >40 (Technical Appendix Table 2). In a multivariate logistic regression model, presence of antibody to the 1918 H1N1 subtype virus was strongly associated with antibody to A(H1N1)pdm09 virus (Technical Appendix Table 3). No demographic factor was independently associated with positivity to A(H1N1)pdm09 virus. By using a nonlinear regression model for the probability of A(H1N1)pdm09 antibody prevalence compared with birth year, we found the model that best fit the age-stratified seroprevalence data inflected near 1927 (Technical Appendix Figure), indicating that persons born before 1927 were most reliably protected.

Our findings show that the prevalence of pandemic influenza virus antibody in a representative population-based 2004 sample of NYC residents correlated with birth year and year(s) of circulating virus. These data reveal the immunologic background during the emergence of A(H1N1)pdm09 virus in NYC beginning in late April 2009 (*7*) and help explain why fewer cases of A(H1N1)pdm09 infection were detected among older persons than younger persons, supporting the conclusion that the difference was a result of, at least in part, antibodies elicited by prior H1N1 subtype infection in older persons.

Viruses antigenically resembling the 1918 pandemic strain circulated among humans earlier in the twentieth century; cross-reactivity with antibodies to those viruses likely provided protection against the 1918 virus. Most (*2*,*4*), but not all (*8*), previous A(H1N1)pdm09 virus seroprevalence studies demonstrated an increase in immunity with age. In our study, more persons born before than after 1927 (i.e., persons >82 vs. those 65–82 years of age in 2009) had HAI assay results positive for A(H1N1)pdm09 virus. Protection among persons 65–82 years old during the 2009 pandemic may be explained by the presence of preexisting immunity not measured by standard HAI tests (e.g., antibodies that target the hemagglutinin stalk) or by T-cell responses (*9*). More positive test results were recorded with the 1918 than the A(H1N1)pdm09 virus; this finding is consistent with the model in which preexisting immunity to A(H1N1)pdm09 virus was derived from exposure to the 1918 pandemic strain or to antigenically related strains that evolved since then (*10*). The 1918 and 2009 strains used in testing may have exhibited different sensitivities in HAI assays. Immunity in older populations is not surprising and was seen in the 1918, 1957, and 1968 pandemics, during which newly introduced pandemic viruses were more likely to cause clinical illness in younger persons, presumably because prior exposure to similar viruses resulted in cross-reactive antibodies (*11*).

Study limitations include a relatively small sample size and a lack of history regarding influenza virus infection or vaccination. Nevertheless, the ability to evaluate seroreactivity in a representative sample of adults helps validate and reinforce previously published findings on H1N1 subtype viruses and clarifies levels of immunity to H2N2 and H3N2 subtype viruses.

Technical Appendix Information on datasets and methods, the characteristics of persons with cross-reactive antibodies to pandemic influenza viruses and different antibody titers to influenza A(H1N1)pdm09 virus, estimates of logistic regression parameters, and the predicted probability of cross-reactivity to influenza A(H1N1)pdm09 virus.
