# Supplementary material for: Seroprevalence of Pandemic Influenza Viruses, New York, New York, USA, 2004
Source: Emerg Infect Dis. 2012 Nov;18(11):1905–7. doi: 10.3201/eid1811.120156 (PMC3559150; doi:10.3201/eid1811.120156)
Supplement: Technical Appendix — Information on datasets and methods, the characteristics of persons with cross-reactive antibodies to pandemic influenza viruses and different antibody titers to influenza A(H1N1)pdm09 virus, estimates of logistic regression parameters, and the predicted probability of cross-reactivity to influenza A(H1N1)pdm09 virus. [file 12-0156-Techapp-s1.pdf]

Article DOI: http://dx.doi.org/10.3201/eid1811.120156

Seroprevalence of Pandemic Influenza Viruses, New York, New York, USA, 2004

Technical Appendix

The New York City Health and Nutrition Examination Survey (NYC HANES) was a population based sample of New York City adults collected in 2004. To determine seroprevalence to 2009 H1N1 antibody, as well as viruses from prior pandemics, a 400 person subsample of repository serum was selected for inclusion in the study, representing persons who were 23 or older in 2004. All specimens were tested by hemagglutination inhibition assays for 1918 H1N1, 1957 H2N2, 1968 H3N2, and 2009 H1N1 pandemic influenza viruses.

Datasets and Methods

NYC Health and Nutrition Examination Survey

In 2004, the New York City Department of Health and Mental Hygiene (NYCDOHMH) conducted a population-based, cross sectional survey of NYC adults modeled after the National Health and Nutrition Examination Survey (HANES). The methodology for NYC HANES has been described in detail elsewhere (*1*). In brief, the survey population included a representative sample of non-institutionalized adults, ages 20 and older, recruited through letters and field visits, followed up by interviews, physical examination and biologic specimen collection for consenting participants. A 3-stage cluster sampling plan was used to recruit participants between June and December 2004. The stages of sample selection were as follows: 1) selection of census blocks or groups of blocks; 2) random selection of households within selected segments; and 3) random selection of study participants within households. The survey included a face-to-face computer-assisted personal interview, private audio computer-assisted self-interview, physical examination, and laboratory testing (sera and urine). The overall response rate was 55%. Participants were asked to consent to allow for collection and storage of additional biologic samples for future research purposes, without notification of the results. Demographic data

collected at the time of interview included age, race/ethnicity, country of birth, and gender. Occupational and influenza histories were not collected.

Specimen Sampling

From the specimen repository we randomly selected serum samples from those persons born in 1981 or earlier (persons age 23 or older at the time of the NYC HANES collection) to include in this study. This would allow us to better understand prior exposure to H1N1 (presumably conferring immunity), which might explain the age-related pattern of illness seen during the 2009 H1N1 pandemic in New York City and elsewhere. Other objectives were to understand the patterns of measurable neutralizing antibody titers to the 1918 H1N1, 1957 H2N2, and 1968 H3N2 pandemic influenza viruses.

The total sample size of NYC HANES was 1,999. Of these, 1,811 completed the serosurvey component. Serum specimens were collected from most consenting participants and are stored at the NYCDOHMH's Public Health Laboratory at –70°C. From these, a subsample of 400 was selected for testing. The data were weighted to adjust for the complex sampling, design nonresponse, and post-stratification. The weights were further adjusted to address component- and item- nonresponse

The subsample was drawn in such a way as to address two distinct purposes of the study. The first was to establish a cutoff birth date, before which individuals may show an increased likelihood of cross-reactivity to the 2009 H1N1 virus . The second was to estimate the level of cross-reactive antibodies for those born before the cutoff. The first purpose favors selecting more samples around the potential cutoff while the second favors selecting sample from respondents born before the cutoff. After establishing a potential range for the cutoff value, the sample was drawn with increasing probability in the range, very low probability below the range, and moderate probability above the range. Component weights were adjusted by their inverse probability of selection into the subsample and then scaled to the population total.

Laboratory Testing

Human Sera Treatment:

Human sera was treated with trypsin periodate, according to established procedures, to remove nonspecific inhibitors of hemagglutination (*2*).

Virus and Virus like particle (VLP) production:

The 1918 pandemic H1N1 and 1957 pandemic H2N2 viruses were assayed using non-infectious virus-like particles (VLPs) rather than live virus to allow for use at biosafety level 1 (BSL1). In brief, VLPs were produced by co-transfecting HEK293T cells with expression plasmids encoding the hemagglutinin (HA) and neuraminidase (NA) corresponding to influenza A/South Carolina/1/18 (H1N1) virus, influenza A/Singapore/1/57 (H2N2) virus or influenza A/Japan/305/57 (H2N2) virus. Supernatants from the transfected cells were harvested 48 hours later, aliquoted and stored at –20°C until use (*3*).

For the pandemic 2009 (H1N1) virus, we utilized a 6:2 PR8 recombinant virus, with the HA and NA of A/California/4/2009 (H1N1) and 6 internal genes from influenza A/Puerto Rico/8/1934 (H1N1) virus *(5)*. This recombinant virus was propagated at 37°C in MDCK cells and could be used at BSL 2 level. The A/HK/1/68 (H3N2) virus was propagated at 37°C in 10 day old embryonated chicken eggs.

Hemagglutination Inhibition (HAI) Assays:

Virus or VLPs were diluted to a concentration of 8–16 HA units/50 mL[3]. The diluted virus or VLPs were incubated with serial two-fold dilutions of trypsin periodate-treated human sera for 30 minutes at 4°C, followed by incubation with 0.5% turkey RBCs for the CA/09 virus and 0.5% chicken RBC's for all the other viruses or VLPs. Results were recorded as the reciprocal of the greatest dilution yielding inhibition of hemagglutination (*4*).

Statistical Analysis

For all analyses a hemagglutination titer of 40 or greater was used to indicate prior exposure and/or immunity to the virus in question. Laboratory data were merged with demographic information including date of birth, age (as of 2004), gender, country of birth (USA vs. foreign born), and race/ethnicity to determine the prevalence of prior exposure by these factors.

Locally weighting smoothing scatterplots (LOESS curves) (*5*) were used to estimate the prevalence of antibodies by year of birth. To appropriately account for the complex survey design of NYC HANES, logistic regression models were fit using Sudaan version 10.0 (*6*). Nonlinear models were used to explicitly estimate the cutoff year for increased probability of immunity, using a segmented regression model that includes cutoff year as a parameter (*7*).

Institutional Review Boards

This study was approved by both the Mount Sinai School of Medicine and NYCDOHMH Institutional Review Boards.

References

1. Thorpe LE, Gwynn RC, Mandel-Ricci J, et al. Study design and participation rates of the New York City Health and Nutrition Survey, 2004. Prev Chronic Dis. 2006;3:1–8.

2. World Health Organization. WHO manual on animal influenza diagnosis and surveillance [cited 2012 Sep 3]. WHO/CDS/CSR/NCS/2002.5Rev.1; p. 37–79. http://www.who.int/csr/resources/publications/influenza/whocdscsrncs20025rev.pdf

3. Yu X Tshidi Tsibane T, Patricia A. McGraw PA, et al. Neutralizing antibodies derived from the B cells of 1918 influenza pandemic survivors. Nature. 2008;455:532–6.

4. Hirst G. K. The quantitative determination of influenza virus and antibodies by means of red cell agglutination. J Exp Med. 1942;75:49–64.

5. Cleveland, WS. Robust locally weighted regression and smoothing scatterplots. J Am Stat Assoc. 1979:74:829–36.

6. Research Triangle International. Research Triangle Park, North Carolina.

7. Muggeo VMR. Estimating regression models with unknown break-points. Stat Med. 2003; 22:3055-71.

Technical Appendix Table 1. Weighted seroprevalence of cross-reactive antibodies to the 1918, 1957, 1968, and 2009 pandemic influenza viruses among persons >23 years of age, New York, New York, USA, 2004*

Characteristic	Total No.	H1N1 (1918)			H2N2 (1957)			H3N2 (1968)			H1N1(2009)		
		No. +	Prev (%)	95% CI	No. +	Prev (%)	95% CI	No. +	Prev (%)	95% CI	No. +	Prev (%)	95% CI
Total	400	24	10.1	6.8–14.8	93	24.2	19.3–29.9	278	72.1	66.9–76.9	17	6.6	4.1–10.4
Age, y													
23–27	31	1	9.4	0.4–17.7	1	1.6	0.2–29.7	10	30.2	16.3–49.1	1	2.8	0.4–17.7
28–32	47	1	5.5	0.2–10.6	3	7.1	2.0–30.3	12	27.0	15.6–42.5	0	0	—
33–37	46	2	3.8	0.9–15.4	1	3.1	0.4–18.5	38	83.0	67.8–91.9	1	2.8	0.4–16.8
38–42	49	0	0	—	2	5.2	1.3–47.8	44	90.9	79.4–96.3	0	0	---
43–47	41	0	0	—	15	40.4	25.6–77.6	36	90.7	76.5–96.7	1	2.6	0.4–17.1
48–52	42	0	0	—	23	59.2	42.0–87.2	37	90.5	78.3–96.1	0	0	—
53–57	35	0	0	—	19	52.5	35.5–89.2	27	79.2	62.0–89.8	1	1.8	0.2–11.6
58–62	26	1	4.8	0.6–28.2	11	42.3	21.7–86.1	21	78.2	53.9–91.7	1	4.8	0.6–28.2
63–67	20	0	0	—	8	37.5	18.2–67.5	13	63.1	36.4–83.6	0	0	—
68–72	14	1	3.3	0.5–18.4	3	15.7	5.2–56.8	9	68.2	43.5–85.6	1	3.3	0.5–18.4
73–77	22	4	20.5	7.5–45.0	3	12.3	3.7–45.1	16	73.5	49.5–88.7	1	4.7	0.6–27.4
78–82	15	8	55.3	31.6–76.8	2	13.8	2.9–65.7	8	60.2	30.5–83.9	3	18.0	5.1–47.1
83–89	12	6	48.5	23.0–74.9	2	19.5	4.8–61.5	7	73.9	41.3–91.9	7	54.2	27.0–79.2
Total for persons ≥65	75	19	29.9	20.1–42.1	15	17.3	10.0–28.5	49	69.4	57.7–79.1	12	17.8	10.3–29.1
Sex													
Male	182	17	14.0	8.6–22.0	44	25.5	18.5–34.2	121	70.2	62.6–76.9	13	10.8	6.4–17.8
Female	218	7	16.3	2.8–13.3	49	22.9	17.0–30.1	157	74.0	66.6–80.3	4	2.3	0.7–7.4
Country of birth													
United States	214	14	11.1	6.6–18.0	55	26.2	19.2–34.5	151	72.5	65.3–78.6	11	7.7	4.2–13.4
Foreign	185	10	8.8	4.5–16.7	38	21.5	15.4–29.2	127	71.9	64.5–78.3	6	5.1	2.2–11.6
Race/Ethnicity													
Non-Hispanic white	147	8	7.8	3.9–14.9	41	28.2	19.8–38.5	120	70.5	61.6–78.0	6	6.0	2.7–12.7
Non-Hispanic black	94	7	14.2	7.0–26.8	24	26.5	18.2–36.9	85	812.	72.5–87.7	5	8.7	3.4–20.6
Non-Hispanic Asian	45	0	0	0	12	23.7	13.7–37.8	38	69.1	52.7–81.8	1	1.7	0.3–10.1
Hispanic	105	9	16.1	8.4–28.7	14	11.5	6.8–18.7	93	66.6	55.4–76.2	5	8.3	3.4–18.9
Non-Hispanic other	8	0	0	—	2	28.1	6.9–67.1	7	68.7	29.8–91.9	0	—	—
Missing	1												

*Prev, prevalence; +, positive; —, no value.

Table 2. Characteristics of persons with different antibody titers to influenza A(H1N1)pdm09 virus, New York, New York, USA, 2004

	Titer		
Characteristic	<20 (n = 362)	20–40 (n = 29)	>40 (n = 9)
Mean age, y	50	72	80
Median age, y	48	79	85
Male, %	48.7	50.3	75.1
US born, %	55.0	74.5	81.3
Hispanic, %	18.4	20.3	27.6

Table 3. Logistic regression parameter estimates for predicted probability of A(H1N1)pdm09 cross reactivity, New York, New York, USA, 2004*

Parameter	Odds ratio	Standard error	95% CI	z score	p value
Intercept	0.002	1.58	–9.31 to -3.12	-3.94	<0.0001
1918 result (positive)	37.317	1.14	1.39–5.85	3.19	0.001
1919 result (negative)	REF	—	—	—	—
Race/ethnicity					
Hispanic	0.843	1.04	–2.22 to1.87	-0.16	0.870
Non-Hispanic Asian	1.709	1.24	–1.89 to 2.96	0.43	0.665
Non-Hispanic black	1.087	0.97	–1.83 to 1.99	0.09	0.932
Non-Hispanic white	REF	—	—	—	—
Country of birth					
US born	1.893	0.80	–0.93 to 2.21	0.79	0.427
Foreign born	REF	—	—	—	—
Year of birth					
1915–1932	2.942	1.10	–1.09 to 3.24	0.98	0.329
1933–1956	1.314	0.77	–1.24 to 1.79	0.35	0.724
1957–1980	REF	—	—	—	—
Sex					
Female	5.081	0.91	–0.15 to 3.41	1,79	0.074
Male	REF	—	—	—	—

*Model treats year of birth as categorical. REF, referent.

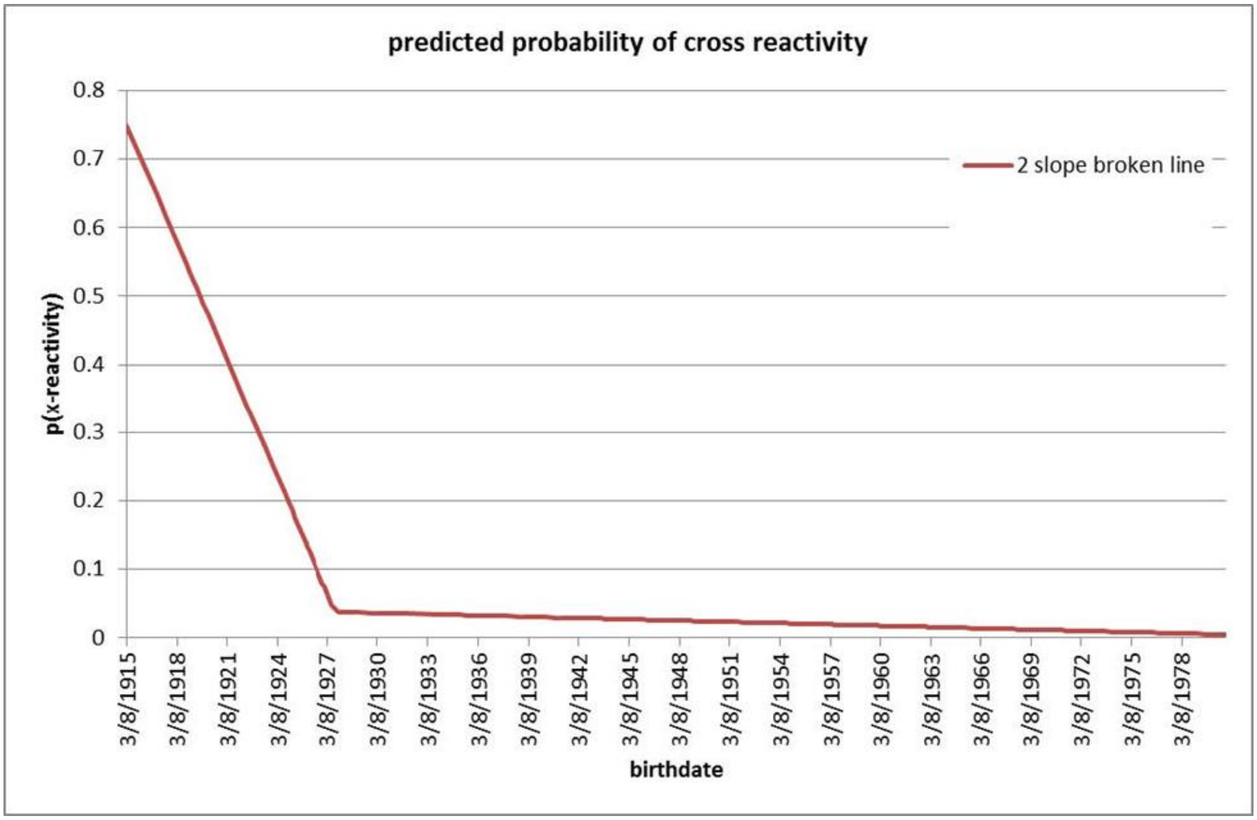

Figure. Predicted probability of cross-reactivity to the influenza A(H1N1)pdm09 virus among persons >23 years of age, New York, New York, USA, 2004. Using a nonlinear regression for the probability of antibody prevalence compared with year of birth, we found that the model that best fits the age-stratified seroprevalence data inflects near birth year of 1927, suggesting a cut-off point for immunity as measured by hemagglutination inhibition assay estimated as July 3, 1927, (95% confidence limits: May 2, 1925, September 3, 1929), and for those after, there is little detectable immunity.
